# Soil Organic Carbon Dynamics and Driving Factors in Typical Cultivated Land on the Karst Plateau

**DOI:** 10.3390/ijerph17165697

**Published:** 2020-08-06

**Authors:** Guandi He, Zhenming Zhang, Jiachun Zhang, Xianfei Huang

**Affiliations:** 1 Institute of Agro-Bioengineering, Key Laboratory of Plant Resources Conservation and Germplasm Innovation in Mountainous Region (Ministry of Education) and College of Life Sciences, Guizhou University, Guiyang 550025, China; heguandi147@126.com; 2 Institute of Biology, Guizhou Academy of Sciences, Guiyang, Guizhou 550009, China; zhangjiachun1988@163.com; 3 Guizhou Provincial Key Laboratory Information System of Mountainous Areas and Protection of Environment, Guizhou Normal University, Guiyang 550001, China; hxfswjs@gznu.edu.cn

**Keywords:** soil organic carbon storage, cultivated land, impact factor, dynamic change, karst

## Abstract

Due to the impacts of unwise industrial agriculture, extreme precipitation events are increasing in frequency and are accelerating the process of global warming in the karst area. The dynamic change in soil organic carbon (SOC) and its driving factors in cultivated land in the last 35 years were studied by using data from the second national soil survey of China and measurements made in 2015. The results indicated that the SOC per unit area of cultivated land increased by 32.45 × 10^3^ t in the last 35 years in the study area, exhibiting basically the same levels and a slight increasing trend, and the annual average change rate was 0.02 kg C·hm^−2^·a^−1^. In terms of spatial distribution, carbon loss areas were mainly concentrated in the middle northern region, western region, and scattered eastern regions of the county. The main factors affecting the change in SOC in the cultivated land in the study area in the last 35 years include nitrogen fertilizer application, stubble, soil thickness, soil total nitrogen, C/N, rock coverage, gravel content, soil organic carbon density (SOCD_1980_), etc. This study will provide a database for the management of SOC in cultivated land in the future.

## 1. Introduction

The soil organic carbon (SOC) pool of cultivated land is the most active part of the global carbon pool, playing an indispensable role in the global carbon cycle [1,2,3]. In addition, SOC is strongly influenced by human activities. Exploring reserve dynamics and change trends of the carbon pool in the region is of great significance for the awareness of the carbon fixation potential of cultivated land and response to climate changes [4,5]. In addition, this research direction provides a scientific reference for cultivated land quality management [6]. There are currently various studies of SOC pools of cultivated land worldwide. For example, Maia et al. performed a positioning experiment to explore SOC changes under different agricultural practices in the southeastern Amazon [7,8]. Minasny et al. found a significant increase in SOC storage in the soil surface (0–15 cm) over three decades of rice growth in Java and Korea using statistical and GIS (Geographic Information System)methods [9]. However, there are few research reports on the SOC storage of cultivated land in karst areas [10]. Current research is mainly concentrated on the effects of certain factors (including different vegetation types, land utility methods, human invention, etc.) on SOC changes in karst areas and on soil organic carbon density (SOCD) and soil property changes in the process of karst rocky desertification [11,12]. Hence, it is necessary to conduct systematic research on SOC dynamics and impacting factors in cultivated land in karst regions, thereby achieving a deep understanding of the carbon fixation mechanisms of cultivated land in karst regions [13].

SOC is affected by many factors, such as physicochemical factors and fertilizers. Some studies have found that a decline in SOC can be stopped through organic manure application and improved nutrient management practices [14]. Hati et al. have investigated the long-term effects of inorganic fertilizer, manure and lime application on the organic carbon content and physical properties of an acidic Alfisol (Typic Haplustalf) under an annual soybean–wheat crop rotation, and they found that soil management practices in acidic Alfisols should include integrated use of mineral fertilizer and organic manure or lime to maintain the organic carbon status and physical environment of soil [15]. Raun et al. have studied the effects of long-term nitrogen application on SOC in continuous winter wheat (*Triticum aestivum* L.) production systems. These authors found that when N was applied at rates >90 kg ha^−1^, the SOC (0–30 cm) was either equal to that of the control (no N applied) or slightly greater, and they concluded that the SOC increased when N was applied at rates in excess of that required for maximum yield [16].

Guizhou Province, with an area of more than 500,000 km^2^, is the center of a mountainous karst area in southwestern China [17]. This area is a typical ecologically fragile region with the largest area and the strongest karst development among the world’s three largest karst-concentrated areas [15]. In addition, this area is one of the regions of China where an extremely impoverished population is relatively concentrated. Because of the special environmental conditions in karst landforms, the agricultural ecological environment is fragile in the karst area, with high ecological environment sensitivity, strong vulnerability, and low stability [18]. Due to the large proportion of sloping cropland, mechanical operation is difficult. To make matters worse, “ecological fragility–poverty–overexploitation–environmental degradation–further poverty” traps the region in the vicious circle known as the “poverty trap”. Puding County, located in the western region of Guizhou Province, is a typical karst plateau region with widely distributed carbonate rocks [19]. Puding County is one of the regions with the strongest karst development in China. The natural environment of Puding County is fragile [20]. Imprudent human activities have made soil erosion and rocky desertification in the region increasingly prominent in recent years. The ecological environment has been seriously damaged, even leading to changes in SOC. In the soil carbon storage system of the region, the most concerning issue is the change in the SOC storage, which is the most active and has the greatest impact on human agricultural production [21]. The karst landform area and rocky desertification area in Puding County are approximately 84.3% and 35.8% of the total area of the county, respectively. The ecology is extremely fragile, and the county features karst landforms typical of Guizhou Province [22]. The aim of this study is to analyze the dynamics and impacting factors of the SOC pool in the surface soil of Puding County over the past 35 years and to reveal the current status of SOC storage in the karst area. Moreover, this study will also provide a scientific reference and a basis for the management of the soil carbon pool of cultivated land in the karst area in the future.

## 2. Materials and Methods

### 2.1. Study Region

**Figure 1:**
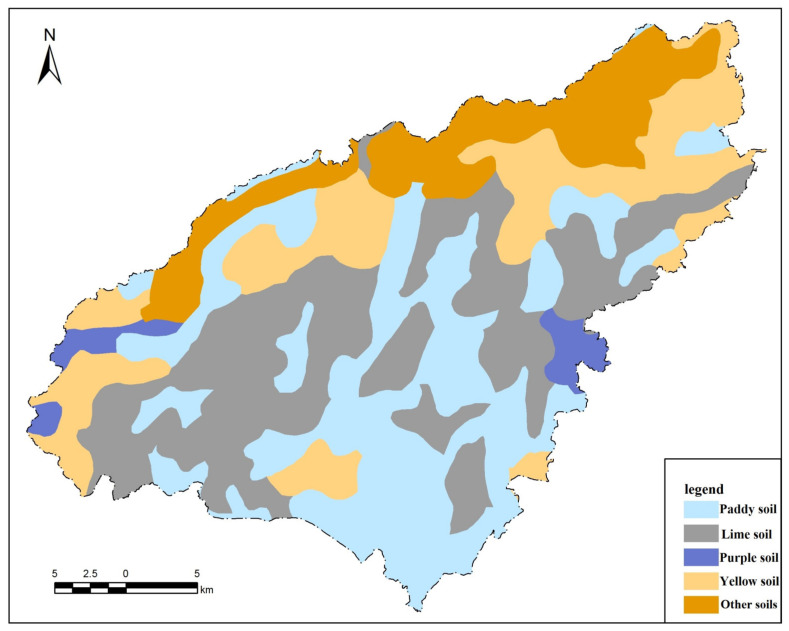
Distribution map of different soil types of study area.

Puding County (105°27′49″–105°58′51″ E, 26°9′36″–26°31′42″ N) is located in the central part of the karst area in western Guizhou Province and east of the Yunnan–Guizhou Plateau. Puding County covers an area of 1079.93 km^2^, and the elevation is 1100–1600 m. Puding County experiences a northern sub-tropical humid monsoon climate. The climate is mild throughout the year, with a long frost-free period, abundant rainfall, less sunshine, and low incident radiative energy. The maximum rainfall is 1465 mm, the minimum is 891 mm, and the average annual precipitation is 1390 mm; under special conditions, the maximum temperature is 34.8 °C, the minimum temperature is −3.4 °C, and the annual average temperature for many years is 15 °C. The topography of Puding County is a typical karst landform, with a wide area of limestone distribution, strong karst development, and abundant features such as depressions, funnels, and caves. The crops are mainly rice, maize, and wheat. Certain economic crops are cultivated at the same time, including rapeseed, peanuts, and flue-cured tobacco. The soil types include moist soil, yellow soil, meadow soil, lime soil, paddy soil, and purple soil [23]. The soil types with the broadest distribution are yellow soil, lime soil and paddy soil, accounting for 37.18%, 33.24% and 27.02% of the total cultivated land area, respectively (Table 1, Figure 1).

### 2.2. Data Sources

**Figure 2:**
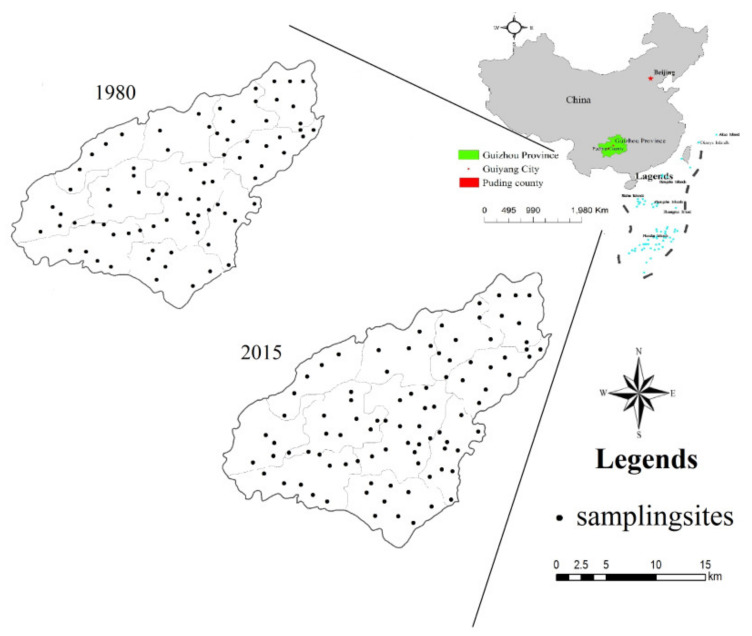
Study area location and soil sampling map of study area.

In this study, the basic information on the soil surface (0–20 cm) was mainly obtained from data for Puding County in the second national soil survey of China (1980) (the data for the SOC contents are for the depth of the A horizon) [24], and actual soil measurements of the cultivated land in Puding County were taken in the winter of 2015 (Table 2), within a period in which no rain had fallen in recent days. The sampling plots for 2015 were determined based on descriptions of sampling positions in the second national soil survey of China in 1980, employing the principle of being as consistent or close as possible. Sample collection was performed according to the S-shape route. Simultaneously, a GPS (Global Positioning System) was used to record information for each sample, such as latitude and longitude, land utilization type, topography, and sampling depth. The topsoil (0–20 cm) was collected as the soil samples. A total of 94 sampling sites were selected (Figure 2). Soil management data for the cultivated land in Puding County in 2015 were obtained from field investigation. Semi-structured interviews of typical farmers were carried out through field interviews and by referencing the county’s statistical yearbook. A semi-structured field survey was performed on the cultivated soil management inputs (data on organic fertilizer use, chemical fertilizer use, crop stubble, straw return and yields of principal crops) by the research team for several months beginning in March 2015. Stubble was mainly estimated from maize on dry land and rice in paddy fields. The application of organic fertilizer mainly involved manures and dung. Dry manure was used with straw return to the field. Chemical fertilizers used were mainly urea, compound fertilizer, and phosphate fertilizer.

### 2.3. Soil Sample Pretreatment and Determination of Physicochemical Properties

During the second national soil survey of China, there were no SOC measurements. The SOC contents were obtained by conversion from soil organic matter to SOC with a conversion coefficient of 1.724. Treatment methods for recent samples were consistent with sample processing during the second national soil survey of China. After mixing, fresh samples were allowed to dry naturally in the room. The dried samples were ground through a 0.2-mm sieve. The SOC content was determined by sulfuric acid-potassium dichromate with external heating. The soil bulk density (SBD) was determined by the cutting-ring method. Each soil sample was removed by a cutting ring, which was repeated at least five times for each soil type. The weight of the cutting ring and the weight of the dry water and the cutting ring were measured. Bulk density for various soil types was calculated by the bulk density calculation formula. Soil thickness was determined by the brazing method under the ecological niche type. Sections of the brazing iron length of 60 cm and 120 cm, suitable for measuring the soil at different depths, were used to determine the average value of the soil thickness at 8–10 sites. The rock coverage was determined by the line-transect method and was expressed as the percentage of area with vegetation coverage and the percentage of rock coverage within each sampling site. The gravel content was expressed as the percentage of the gravel volume greater than 2 mm. The Agrochemical Soil Analysis Method was used as a reference for the determination of the chemical element contents and other parameters, including total N, total P, total K, effective P, rapidly available K, soil pH, etc. [25]. The results of soil property measurements are shown in Table 2.

### 2.4. Calculation of Soil Organic Carbon Density

The formula for the soil organic carbon density (*SOCD*) *SOCD_j_* (kg C·hm^−2^) of a certain soil was [11]:

(1) $$SOCD_{j}=C_{j}\times D_{j}\times E_{j}\times (1-G_{j})/100$$

where $C_{j}$ is the SOC content (%), $D_{j}$ is the bulk density (g·cm^3^), $E_{j}$ is the soil thickness (cm), and $G_{j}$ is the percentage of the gravel volume larger than 2 mm. The SOC stock (*Tj*) of a soil type is the product of the SOC density (*SOCD_j_*) of the soil at a 0–20 cm depth and its distribution area (*Sj*); that is, *Tj* = (*SOCD_j_* × *Sj*)/1000. Where the unit of *Tj* is *t*, the unit of *SOCD_j_* is kg C·hm^−2^, and the unit of *Sj* is hm^2^.

### 2.5. Data Processing

The area of cultivated land in Puding County has changed over the past 35 years (1980–2015) with the influence of land utilization change and land use structure adjustment. In this study, the impact of soil change on the SOC was considered. Interconversion of non-cultivated land and cultivated land was not considered. In accordance with the relevant collected data and maps, the areas of cultivated land occurring in the soil utilization maps of Puding County from both 1980 and 2015 were selected to comprise the cultivated land range of this study. ArcGIS software was used for the superposition of the selected cultivated land map and soil type map of Puding County, thereby obtaining the cultivated soil map of Puding County. Assuming the same SOC dynamics and soil physicochemical properties for the same soil, values were assigned to various soil types to obtain spatial distribution maps of SOC dynamics and soil physicochemical properties. The spatial distribution map was transformed into 90 m × 90 m-resolution grid maps using ArcGIS software. The grid map was converted into a point file. Microsoft Excel was used to derive the coordinates of each grid centre point, thereby obtaining the spatial attribute values for SOC dynamics and physicochemical properties [26].

### 2.6. Selection of Factors Affecting SOC Change in Cultivated Land

There are many factors affecting soil properties, with strong multiple collinearities. Especially when there is high dependence among many variables, stepwise regression analysis (SRA) should be used to exclude the variables with insignificant influence. The variables passing the significance testing are sorted by standardized coefficients. Therefore, SRA was used in this study to explore the drivers that influence the SOC changes within the study area and to clarify the order of importance. Changes in the SOCD in the study area between 1980 and 2015 were used as the dependent variables, and the factors described above were used as independent variables. In the process of SRA, the significance of the impact of each independent variable on a particular dependent variable were determined sequentially. If not significant, an independent variable was excluded, and SRA was repeatedly performed in this manner, until the last independent variable. Finally, the stepwise regression equation of SOCD on the impacting factors was obtained.

The main five factor groups affecting the SOC dynamics of the study area in this research are shown in Table 1. The first factor group is the 1980 SOCD background, comprising the initial values of soil physicochemical properties in the study area. The initial values directly affect the changes in SOC. The SOCD in 1980 is one of the first-choice factors for analysing the SOC dynamics in the research area over the past 35 years.

The second factor group is the terrain. In mountainous terrain, the topographic relief greatly influences the differentiation of soil physicochemical properties in the study area, especially the slope gradient and elevation. In addition, the slope position, slope direction, rock coverage, and soil thickness greatly affect the intervention of human activities.

The third factor group includes the soil physicochemical properties. SOC is significantly influenced by physicochemical properties, such as soil bulk density, gravel content, soil water content, etc. With increasing bulk density and gravel content, SOC content decreases. Chemical properties such as effective P, rapidly available K, total N, total P, total K, and soil C/N positively impact SOC. Therefore, for soil physicochemical properties, soil bulk density, gravel content, soil texture, effective P, rapidly available K, total N, total P, total K, pH, and C/N in cultivated land were selected as factors influencing SOC changes in the study area.

The fourth factor group is fertilization. Applying organic fertilizer greatly increases the SOC content. The application of chemical fertilizers (P, N, and K) increases the nutrient elements in the soil, which has a certain effect on SOC. Based on these considerations, organic fertilizers, N, P, and K were selected as the fertilization factors.

The fifth group comprises the biological factors. Yield, stubble, and straw return are the most important biological factors in the study area. Crops grow well with high yield. There is much rhizome underground biomass and litter. Rhizomes and litter function similarly to stubble and straw return, directly increasing organic matter in the soil, thus increasing the SOC content. Hence, the yield, stubble, and straw return are the biological factors that affect the SOC content of the study area.

### 2.7. Stepwise Regression Analysis (SRA) and Redundancy Analysis

There are many factors affecting the changes in SOC in cultivated land, and different factors may be related to each other. To explore the main driving factors affecting the change in SOC in the cultivated land in Puding County from 1980 to 2015, SPSS 18.0 statistical software was used to perform SRA. The annual variation in the SOC density (kg C·hm^−2^·a^−1^) was used as the dependent variable. The indicators listed in Table 3 were the independent variables. The significance of the effects of independent variables on the dependent variables was determined. The indicators with low significance were removed. The equation for regression of annual variation in SOCD on influencing factors was finally obtained. Correlations between organic carbon and topographic factors were analysed using the hmisc package of R (version 3.6.0) within the R Studio platform. The spatial distribution map of organic carbon density and storage was drawn by kriging interpolation in ArcGIS 9.3 software. Redundancy analysis (RDA) is usually used to analyse soil characteristics and correlations with environmental factors.

Models with a 2:1 training-to-test-data split were implemented. The data from the study area were randomly divided into a training data set and an independent verification data set. We used the training set to perform 10-fold cross-validation to test different models with up to 20 rules in each. We used a range of statistics to assess the quality of the predictions. The Pearson correlation coefficient (R2) was used to assess variation and correspondence between the predictions and original data, the root mean squared error (RMSE) was used to quantify the inaccuracy of the predictions, the mean error (ME) was used to assess bias, and finally, the standard deviation of Moran’s I (Z) was used to assess the precision of the predictions [26].

## 3. Results and Analysis

### 3.1. SOCD Changes in the Cultivated Land on the Karst Plateau in the Last 35 Years

The SOCD of the 0–20 cm soil layer in the karst plateau has increased slightly over the past 35 years, with an overall increase of 1.22% (Table 4). Table 4 shows that the SOC density of paddy soil increased the most over the past 35 years. Yellow soil in dry land increased by 15.98%. The organic carbon density decreased for the other soil types. The annual average change in SOCD on the surface of cultivated land in the karst plateau from 1980 to 2015 was 0.02 kg C·hm^−2^·a^−1^. Among the changes, the annual increase in paddy soil was 0.56 kg C·hm^−2^·a^−1^, accounting for the greatest increase, followed by yellow soil, with an increase of 0.17 kg C·hm^−2^·a^−1^. Carbon storage decreased in meadow soil, lime soil, and purple soil. The largest decline occurred in meadow soil, with a decrease of 3.49 kg C·hm^−2^·a^−1^. Although the average annual reduction in SOCD was much greater than the annual increase, the sum of the area of paddy soil, yellow soil and moist soil (35,532.45 hm^2^) was approximately 1.8 times than that of the sum of the other soil types (19,739.39 hm^2^), and thus, the soil types with large areas dominated the change in the cultivated land carbon pool for the whole study area. Therefore, the SOCD of the cultivated land on the karst plateau shows a slight increase in general. Taken together, the accumulation of SOC in the paddy fields is significantly higher than that in the dryland soil. This observation may result from the damage to stability of soil aggregate structure in cultivated land with human intervention and the weakness of the physical protection of SOC. However, paddy fields under waterlogged conditions inhibit the mineralization of SOC to a certain extent and are thus more conducive to the accumulation of SOC than is dry land.

**Figure 3:**
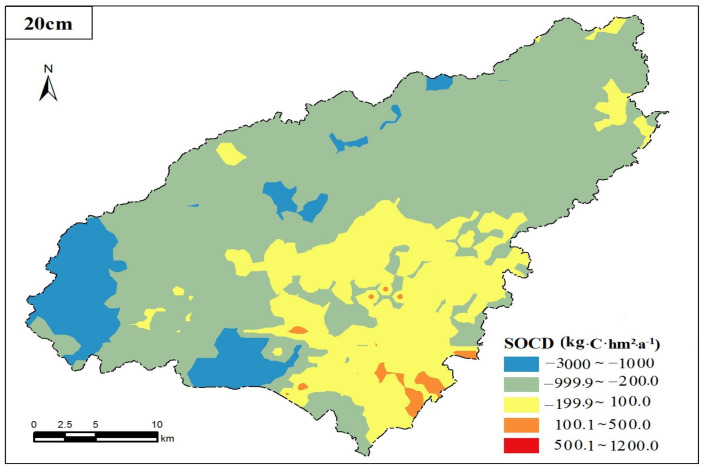
Spatial distribution pattern of annual variation of SOCD in surface soil of farmland in Puding County from 1980 to 2015.

Figure 3 indicates that the average annual changes in the SOCD in the surface soil of the karst plateau showed an increasing trend in the southern, central and northern Sancha River and the surrounding Yelang Lake areas and showed a decreasing trend in most of the western, southern, small portions of central and northern areas of the county. The main reason for this difference in spatial distribution is the varying distributions of soil types. There is abundant paddy soil in the central and southern portions of the study area and the area near the Sancha River. Therefore, the advantages of paddy soil for carbon storage should be fully expressed as carbon accumulates in Puding County. Additionally, reasonable cultivated land management measures for soil types with low SOCD should be selected to improve the SOCD of the cultivated land in Puding County.

The optimal semi-variance function and corresponding parameters of soil SOCD in two periods in the study area are shown in Table 5. In terms of fitting accuracy, the standard deviation of the residual error was close to 0, and the coefficient of determination R^2^ was close to 1, which indicated that the semivariogram fitted in this study was able to reflect the spatial structure characteristics of soil SOCD. The optimal theoretical model of the semi-variance function of soil SOCD in 1980 and 2015 is the Gaussian model. The C_0_/C_0_ + C of soil SOCD in the two periods ranged from 0.50 to 0.75, indicating moderate spatial correlation. Moreover, the standardized Z values of Moran’s I in the two periods were greater than 2.58, indicating significant spatial autocorrelation for both (*p* < 0.01), and the spatial aggregation characteristics were obvious.

### 3.2. SOC Storage Changes in Cultivated Land of Karst Plateau in Recent 35 Years

The research indicated that the SOC storage of the soil surface (0–20 cm) in cultivated land in the study area increased slightly. SOC storage increased from 2682.50 × 10^3^ t in 1980 to 2714.95 × 10^3^ t in 2015, with an overall increase of 1.21%. The soil of cultivated land in the county showed a weak carbon sink increase effect in the past 35 years. In Table 6, it demonstrated that the largest increase in SOC storage was paddy soil (ΔT = 291.16 × 10^3^ t) between the different soil types, with an increase of 38.50%, followed by yellow soil (ΔT = 97.77 × 10^3^ t), with an increase of 12.66%, and SOC storage in lime soil showed the greatest drop (ΔT = −320.78 × 10^3^ t). Thus, the increase of SOC storage of the soil surface on cultivated land in karst plateau from 1980 to 2015 is mainly related to the increase of organic carbon storage in paddy soil and yellow soil. Taking the ratio of SOC storage change from 1980 to 2015 to the SOC1980 reserves as the variation in cultivated land in the study area, it shows carbon loss when the negative variation is greater than 5%; carbon fixation for the positive variation greater than 5%; and relative balance for the variation between ± 5%. In Table 6, it showed that the ratio of carbon loss, carbon fixation and relative balance area in 0–20 cm soil surface in the past 35 years was 26.12%, 61.09% and 12.79%, respectively. In which, the meadow soil and lime soil had the most serious carbon loss, with the loss of 73.06% and 29.77%, respectively. The suggesting that effective reduction of the organic carbon loss in meadow soil of mountain bushes and yellow-brown soil in mountainous regions is a key entry point for improving SOC storage of cultivated land in karst plateau.

**Figure 4:**
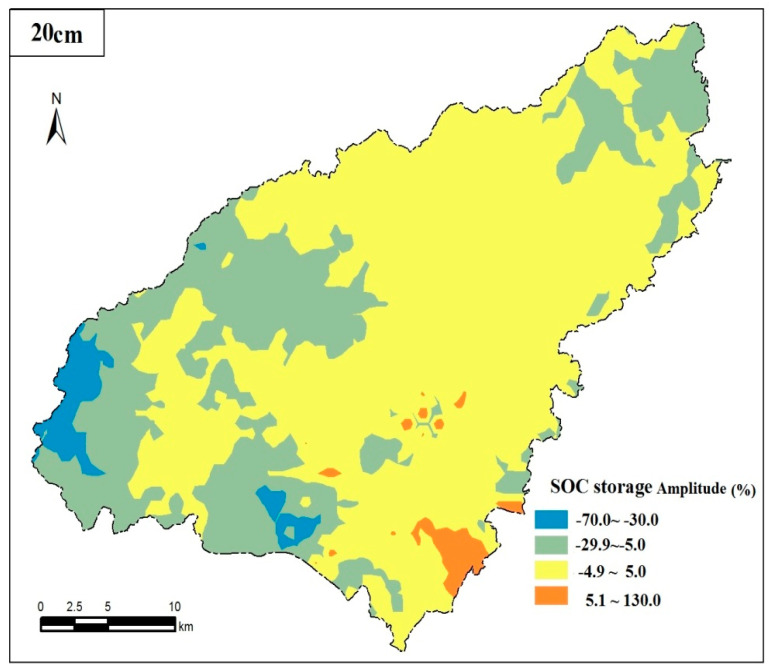
Spatial distribution pattern of SOC storage in surface layer of cultivated land in Puding County from 1980 to 2015.

In the past 35 years, the spatial distribution difference of SOC storage in cultivated land is significant (Figure 4). The carbon loss (with the loss greater than 5%) soil is mainly concentrated in the northern, western and eastern spotted areas of the county. Carbon fixation (with the increase of greater than 5%) soil is mainly concentrated in the banks of the Sancha River, the edge of Yelang Lake, and the central and southern parts of the county. In addition, soil with relatively balanced SOC storage (variation at the range of ±5%) is mainly distributed in the southeast and most part of the central region. Soil properties are good in areas with the increase of organic carbon storage. In addition to human activity influence, human soil utility practices have a critical impact on the changes of SOC storage in the study area, especially in the aspects of fertilization, straw returning, and stubble. Field surveys and interviews suggest that stubble in paddy fields (paddy soil) is the most common activity in the study area. The application of chemical fertilizers and organic fertilizers in paddy soil is greater than that in other soil types, which is conducive to the increase of SOC storage at a certain extent. Farming conditions in areas with decreased organic carbon storage are poor and agricultural inputs are limited, causing a small variation in SOC storage and even a carbon loss. In general, it shows a very slight carbon fixation in the study area.

### 3.3. Factors Impacting SOC Changes of Cultivated Land in the Study Area

#### 3.3.1. Topographic Factors Affecting SOC Content in Cultivated Land on the Karst Plateau

**Figure 5:**
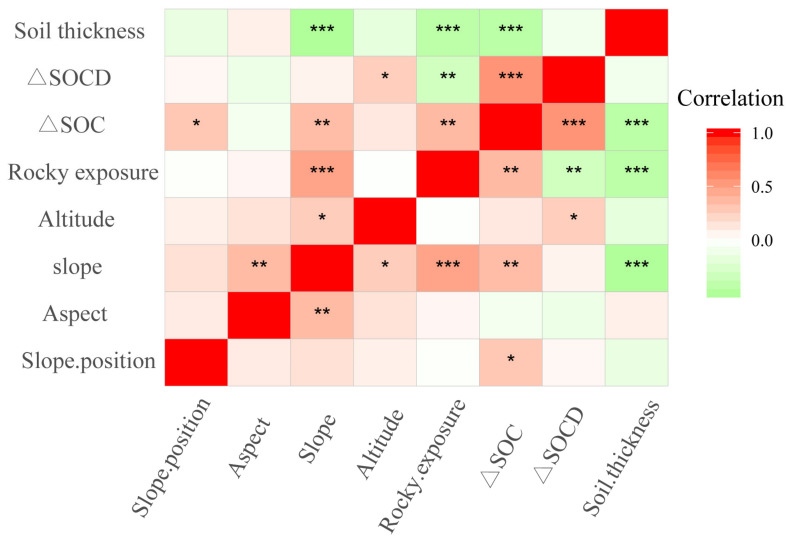
Two-dimensional ordination map of soil organic carbon and topographic factors in cultivated land of Karst Plateau. (”*” represents = 0.01–0.05 (*p* value), ”**” represents 0.001–0.01 (*p* value) and ”***” represents < 0.001 (*p* value))

The results from the correlation analysis are presented in Figure 5. The redder the colour in a square is, the greater the relationship between the two indicators is. The Pearson correlation coefficient was used in this study to reflect the degree of linear correlation between two variables, and its value ranged from −1 to 1. When the linear relationship between the two variables increases, the correlation coefficient approaches 1 or −1. When one variable increases with another variable, there is a positive correlation between them, and the correlation coefficient is greater than 0. When one variable decreases with another variable, there is a negative correlation between them, and the correlation coefficient is less than 0. If the correlation coefficient is equal to 0, there is no linear correlation between them. As shown in Figure 4, the change in SOC was significantly positively correlated with SOCD, rock exposure and slope. The change in SOCD was significantly positively correlated with elevation. The change in SOC was significantly negatively correlated with the soil thickness, which reflects that SOC content decreases with increasing soil thickness. The value of correlation between the change in SOCD and rocky exposure was 0.001–0.01, indicating significant negative correlation.

#### 3.3.2. Key Factors Affecting the Change of SOCD in Karst Cultivated Land

Annual average rates of SOCD change in cultivated land in the study area were used dependent variables and the selected impact factors (24 indicators) in Table 1 were used as independent variables to establish the SRA model. It obtained: ΔSOCD/*t* = −3607.54 + 0.34 *X*_41_ − 357.03 *X*_32_ + 67.44 *X*_46_ + 0.01 *X*_43_ + 0.12 *X*_23_ + 0.45 *X*_52_ − 0.65 *X*_54_ − 0.03 *X*_45_ + 32.12 *X*_47_ + 0.36 *X*_62_ − 0.23 *X*_53_ − 0.02 *X*_51_ − 0.09 *X*_61_ − 123.45 *X*_31_ + 0.07 *X*_42_ − 0.03 *X*_11_ + 25.32 *X*_26_ − 654.21 *X*_25_ + 0.03 *X*_21_ + 0.05 *X*_22_. The overall fitting effect of the model was good, *R*2 = 0.98 (Table 7). In accordance with the standard regression coefficient of variables, it is preliminarily considered that nitrogen fertilizer application, stubble, soil thickness, soil total nitrogen, C/N of cultivated land, rock coverage, gravel content, SOCD_1980_ and other indicators are the key factors affecting the SOC change of cultivated land in the study area in recent 35 years. In addition, from the development direction of the fitting coefficient, it is a positive correlation between nitrogen fertilizer application, stubble, soil thickness, total nitrogen, C/N of cultivated land, and an annual average rate of SOCD change. The other three factors are negatively correlated.

## 4. Discussion

### 4.1. Effects of Topographic Factors on SOC Content Changes in Karst Cultivated Land

The special physicochemical properties and the terrain features in carbonate rock areas result in shallow soil in the karst area and high rock coverage [26]. The soil is often divided into discontinuous patches [27]. SOC presents high spatial heterogeneity. Puding County is a typical plateau karst area with complex and diverse topography spatial distribution [28]. A shallow layer of soil, high rock coverage, and discontinuous soil mantle in this area lead to a great negative correlation between rock coverage, gravel content, and SOC content in karst areas [29]. SOC content in the cultivated land is in the fragmented spatial distribution in general. SOC content showed an increasing trend in the southern, central and northern Sancha River and surrounding Yelang Lake areas, whereas showed a decreasing trend in the majority of western, southern, small parts of central and northern parts of the county [30]. The reason is that peak forests, the peak clusters and the small depression in most of the western parts, the southern, and the central and northern areas of the county are distributed crossly. The soil thickness of the peak forests and peak clusters is shallow and SOC is easy to be lost. However, depression is mainly distributed in the southern, central and northern regions of the study area. The layer of soil is thick and the SOC content is high in the regions. Therefore, the distribution characteristics of SOC in cultivated land in the study area are basically in agreement with the complex topographical characteristics in the basin.

The SOC content is in the fragmented distribution in the karst area with the characteristics of the shallow layer of soil, discontinuous soil mantle, cross-distribution of various soil types, and diverse soil thickness [31]. The rock coverage and the gravel content not only affect the soil thickness but also indirectly affect the soil mechanical composition [32]. The content of silt and clay content is low in the area with high gravel content [33]. The mechanical composition of soil directly affects the change of SOC storage and density. The reduction of the ratio of clay particles decreases the SOC mineralization in the cultivated land in the study area, thereby accelerating the release of SOC in the surface of the cultivated land. In other words, high content of clay particles indicates large SOC storage in the cultivated land and vice versa. This is consistent with the results of Tiessen et al. [34] obtained from the study on soil organic matter and particle composition relationship. In addition, Martel et al. also think that soil clay plays a certain role in SOC protection [35]. With the topographic relief limitation of the cultivated land in the study area, sloping cultivated land is the main form, especially in the lime soil and yellow soil area. Hence, arable land cultivation is subject to the mismatch of water and soil resources, and the moisture holding capacity of the plow layer is decreased. SOC content presents different degrees of change in particular of the limitation of frequent local climate fluctuations.

### 4.2. Effects of Human Activities and Nitrogen on SOC Content Changes in Karst Cultivated Land

The effects of soil total nitrogen and C/N of cultivated land on SOC content of cultivated land in the past 35 years are significantly positively correlated, with standardized coefficients of 0.38 and 0.41, respectively. Soil total nitrogen and C/N, regarded as key indicators for the nutrient balance measurement of C and N, have a great influence on the cycle of SOC. Without any doubt, the factors, affecting the change of total N density, certainly have impacts on soil-cultivated land, thereby acting in mineralization volume and the mineralization rate of SOC accumulation. Qin et al. obtained similar results from the analysis of regional differences in soil carbon-nitrogen coupling characteristics of cultivated land in China [36]. The reduction of soil nitrogen content in the study area increases the consumed carbon for microbial respiration, resulting in the increase of the decomposition and mineralization rate of SOC and the accelerated release of CO_2_ from the soil. SOC is decreased with the reduction of cultivated land. Perez et al. obtained similar results. Furthermore, a faster accumulation and decomposition rate of SOC than that of soil total nitrogen results in the lower effect of decreased cultivated land on SOC than that of soil total nitrogen density. Paddy soil and purple soil in the study area are planted with rice and rapeseed. Lime soil and yellow soil are mainly used for the cultivation of corn and tobacco. The planting area of the former in 2015 was decreased than that in 1980 because of the young adult labor force migration. The planting of the latter was decreased than that in 1980 in spite of little change in planting. The utility change in the cultivated land results in various degrees of the drop in the total N. A drop in the total N density results in the decrease of SOC content. SOCD is on the rise due to rich mineral nutrients in paddy soil, high fertility of soil, and a large volume of organic matter input into the soil in the process of human utilization. The shrub meadow soil is the main distribution in most of the high-altitude northern Doupeng Mountain. The original vegetation in the early 1980s was weeds and shrubs, with high soil humification and the abundant organic matter. After approximately 35-year cultivation, the undisturbed soil was destroyed and the soil organic matter content decreased, leading to the decrease of the organic carbon density.

Since the implementation of the production contract responsibility system in each region in the 1980s, the increase in surface SOC storage may be affected by the increased total agricultural yield and the cultivated land management (stalk returning, increasing application of organic fertilizer, etc.) in China in recent years [37,38]. With the increase of net primary productivity of cultivated land, the total biomass of crops also enhances, thereby increasing the total amount of straw returning [39]. Therefore, the input of soil organic matter is on the rise. The development and utilization of cultivated land have a long history of the agricultural areas in southern China. Suitable hydrothermal conditions enable double or even triple cropping mode in most cultivated land. The cultivated land is developed and utilized with high intensity although certain management measures increase the surface SOC storage. The direct sources of organic carbon in cultivated soil are comprised of ground straw, underground roots, the secretion and shed tissue cells in the growth of root, as well as organic fertilizers. The input of chemical fertilizers in China’s cultivated land has continuously increased in the past 35 years, whereas the application of organic fertilizer is insufficient. Therefore, the returning of crop residues is particularly significant for maintaining or increasing the SOC content in cultivated land.

## 5. Conclusions

The SOCD of 0–20 cm layer of cultivated soil in the karst plateau has increased slightly in the past 35 years, with an overall increase of 1.22%. The storage of meadow soil, lime soil, and purple soil all showed a decrease. The ratio of carbon loss, carbon fixation and relative balance area in 0–20 cm soil surface in the past 35 years was 26.12%, 61.09%, and 12.79%, respectively. In which the meadow soil and lime soil had the most serious carbon loss, with the loss of 73.06% and 29.77%, respectively. In the past 35 years, the spatial distribution difference of SOC storage in cultivated land was significant. Carbon-loss soil is mainly concentrated in the northern, western and eastern spotted areas of the county. Carbon fixation area is mainly concentrated in the banks of the Sancha River, the edge of Yelang Lake, and the central and southern parts of the county. SOC and SOCD are in significantly positive correlation with rock coverage and slope. Nitrogen fertilizer application, stubble, soil thickness, soil total nitrogen, C/N of cultivated land, rock coverage, gravel content, SOCD_1980_ and other indicators are the key factors affecting the SOC change of cultivated land in the study area in recent 35 years. There is a positive correlation between nitrogen fertilizer application, stubble, soil thickness, total nitrogen, C/N of cultivated land, and an annual average rate of SOCD change. The other three factors are negatively correlated.

## Figures and Tables

**Table 1:** Distribution of Soil Area and Survey Sample Statistics in the Study Area.

Soil Types	Areas/hm^2^	Sampling Numbers in 1980	Sampling Numbers in 2015
moisture soil	44.08	3	3
yellow soil	20,551.53	28	30
meadow soil	17.71	3	3
lime soil	18,373.89	13	25
paddy soil	14,936.84	31	26
purple soil	1347.79	5	7
Total	55,271.84	83	94

**Table 2:** Statistics of measured values of basic soil properties in surface layer (0~20 cm) of cultivated land in Puding County in 2015.

Soil Types	Lands	pH	SOC	Total N	Total P	Total K	Effective P	Rapidly Available K	Soil Bulk Density	Gravel Content	Soil Water Content
moisture soil	dry fields	6.6–6.8	25.21–26.34	2.12–2.58	2.61–2.89	21.11–22.12	15.21–16.02	167.98–192.12	1.12–1.15	3.21–3.67	14.12–16.56
yellow soil	dry fields	4.3–8.2	14.12–48.76	1.17–3.43	1.17–3.38	5.67–49.97	1.45–32.12	41.67–267.32	0.91–1.27	1.12–18.73	12.12–37.12
meadow soil	dry fields	6.2–6.7	27.12–28.76	2.47–2.87	1.84–2.17	15.71–16.72	11.12–12.34	122.32–129.76	1.02–1.10	4.32–6.78	16.76–21.23
lime soil	dry fields	5.5–8.3	7.78–39.82	1.01–3.56	1.47–4.78	9.11–51.12	4.76–27.76	54.21–675.32	0.82–1.28	8.98–17.65	18.12–40.08
purple soil	dry fields	4.8–7.5	14.87–29.13	1.26–1.93	1.69–1.97	11.87–20.11	8.32–17.12	102.21–138.66	1.07–1.22	3.09–18.72	15.12–36.56
paddy soil	paddy fields	4.8–8.1	10.21–68.89	1.22–4.67	1.56–5.51	10.12–43.23	7.67–78.87	67.98–667.21	0.83–1.23	1.12–3.23	12.12–15.23

**Table 3:** Main factors affecting soil organic carbon density (SOCD) change rate of cultivated land in the study area.

Category	Index	Number
The 1980 SOCD background (X_1_)	SOCD_1980_ *X_11_*	1
Terrain factor (X_2_)	Elevation (*X_21_*), slope gradient (*X_22_*), slope position (*X_23_*), slope direction (*X_24_*), rock coverage (*X_25_*), soil thickness (*X_26_*)	6
Soil physical index (X_3_)	Gravel content (*X_31_*), soil bulk density (*X_32_*), soil water content (*X_33_*)	3
Soil chemical index (X_4_)	Total N (*X_41_*), total P (*X_42_*), total K (*X_43_*), effective P (*X_44_*), quick available K (*X_45_*), soil C/N (*X_46_*), soil pH (*X_47_*)	7
Fertilization factor (X_5_)	Applying organic fertilizer (*X_51_*), Applying Nitrogenous fertilizer (*X_52_*), Applying phosphate fertilizer (*X_53_*), Applying potash fertilizer (*X_54_*)	4
Biological factor (X_6_)	stubble (*X_61_*), straw returning (*X_62_*), Straw yield (*X_63_*)	3

**Table 4:** Changes of organic carbon density in surface soil of farmland in Puding County from 1980 to 2015.

Soil Types	Areas/hm^2^	SOCD_1980_	SOCD_2015_	∆SOCD	SOCD Amplitude (%)	SOCD/t (kg C·hm^−^^2^ y^−1^)
		(×10^3^ kg C·hm^−^^2^)				
moisture soil	44.08	49.05	50.12	1.07	2.18	0.31
yellow soil	20,551.53	36.49	42.32	5.83	15.98	0.17
meadow soil	17.71	167.40	45.32	−122.08	−72.93	−3.49
lime soil	18,373.89	58.65	41.19	−17.46	−29.77	−0.50
paddy soil	14,936.84	50.63	70.12	19.49	38.49	0.56
purple soil	1347.79	53.12	41.89	−11.23	−21.14	−0.32
Total	55,271.84	48.53	49.12	0.59	1.22	0.02

Note: SOCD_1980_ is the topsoil (0~20 cm) soil organic carbon density in 1980; SOCD_2015_ is the topsoil (0~20 cm) soil organic carbon density in 2015; ΔSOCD is the topsoil (0~20 cm) soil carbon increment per hm^2^; ΔSOCD/t is annual variation of topsoil soil (0~20 cm) soil organic carbon density.

**Table 5:** Semivariogram model of SOCD and its parameter values.

*Time*	*Model Type*	*Nugget (C_0_)*	*Sill (C_0_ + C_1_)*	*C_0_/C_0_ + C*	*Range (m)*	*R^2^*	*RMSE*	*Fractal Dimension (FD)*	*Moran’s I*	*Z*
1980	Gaussian	0.17	0.25	0.68	2831.6	0.96	1.25 × 10^−5^	1.84	0.21	4.17
2015	Gaussian	0.31	0.49	0.63	2926.3	0.93	1.15 × 10^−5^	1.91	0.25	4.05

**Table 6:** Changes of SOC storage in surface soil of farmland in Puding County from 1980 to 2015.

Soil Types	Areas/hm^2^	T1980/(×10^3^ t)	T2015/(×10^3^ t)	ΔT/(×10^3^ t)	ΔT/T1980/%	Lost Carbon (%)	Fixed Carbon (%)	Relative Balance (%)
moisture soil	44.08	2.16	2.21	0.05	2.31	15.00	5.00	80.00
yellow soil	20,551.53	771.97	869.74	97.77	12.66	17.83	80.19	1.98
meadow soil	17.71	2.97	0.80	−2.17	−73.06	85.00	3.00	17.00
lime soil	18,373.89	1077.60	756.82	−320.78	−29.77	82.00	3.00	15.00
paddy soil	14,936.84	756.21	1047.37	291.16	38.50	12.16	49.76	38.08
purple soil	1347.79	71.59	56.46	−15.13	−21.13	83.11	14.12	2.77
Total	55,271.84	2682.50	2714.95	32.45	1.21	26.12	61.09	12.79

Note: T1980, T2015 are the SOCS of the surface layer of 1980 and 2015; ΔT is the SOCS change of the surface layer; ΔT/T1980 is the ratio of soil organic carbon storage to the carbon storage in 1980; lost carbon, fixed carbon and relative balance indicate that the SOCS amplitude is less than −5%, greater than 5% and between 3 and 5% in 1980–2015 years respectively.

**Table 7:** Principal Component Analysis of Influencing Factors of Soil Organic Carbon Density.

	Non-Standardization Coefficient		Standard Coefficient
Variable	B	Standard Error	
Variable	−3607.54	12.56	-
*X*_11_	−0.03	0.00	−0.89
*X*_41_	0.34	0.00	0.38
*X*_46_	67.44	0.09	0.41
*X*_43_	0.01	0.00	−0.02
*X*_31_	−123.45	1.87	−0.71
*X_47_*	32.12	0.18	0.06
*X_42_*	0.07	0.00	0.13
*X_45_*	−0.03	0.01	−0.02
*X_52_*	0.45	0.54	0.06
*X_53_*	−0.23	0.06	−0.01
*X_61_*	−0.09	0.49	−0.02
*X_51_*	−0.02	0.00	−0.02
*X_62_*	0.36	0.02	0.02
*X_54_*	−0.65	0.07	−0.03
*X_32_*	−357.03	1.68	−0.06
*X_26_*	25.32	0.02	0.53
*X_25_*	−654.21	1.76	−0.43
*X_21_*	0.03	0.01	0.02
*X_22_*	0.05	0.03	0.00
*X_23_*	0.12	0.04	0.00
